# Effectiveness of Banxia Xiexin Decoction in the treatment of precancerous lesions: A protocol for systematic review and meta-analysis

**DOI:** 10.1097/MD.0000000000025607

**Published:** 2021-04-23

**Authors:** Ying Yi, Ziyi Hu, Renliang Li, Lisha Chen, Hengyi Zhang, Hong Li, Mengwen Wu, Wanting Liu

**Affiliations:** a Jiangxi University of Traditional Chinese Medicine; b The Affiliated Hospital of Jiangxi University of Traditional Chinese Medicine, Nanchang, China.

**Keywords:** Banxia Xiexin Decoction, chronic atrophic gastritis, gastric precancerous lesions, protocol

## Abstract

**Background:**

Gastric precancerous lesion (GPL) is a necessary stage in the occurrence and development of gastric cancer. At present, the incidence of gastric cancer is increasing year by year. It is important to prevent and control gastric cancer through early detection and intervention. Banxia Xiexin Decoction (BXD) has a good effect in improving symptoms, reducing inflammation, promoting the repair of gastric mucosa, reversing its atrophy and intestinal metaplasia. BXD may be a foreground choice for the treatment of GPL.

**Methods:**

Randomized controlled trials of BXD for GPL will be searched in the relevant database, including PubMed, Embase, Cochrane Library, China National Knowledge Infrastructure (CNKI), Wanfang Database, Chinese Biomedical Literature Database (CBM), and Chinese Scientific Journal Database (VIP database). The studies of electronic searches will be exported to EndNote V.9.1 software. We will run meta-analyses using the Review Manager (RevMan) V.5.3 software. Any disagreement will be solved in consultation with a third reviewer.

**Results:**

Our study aims to explore the efficacy of BXD for GPL and to provide up-to-date evidence for clinical of GPL.

**Conclusion:**

The conclusion of this study will provide evidence for the efficacy of BXD on GPL.

**INPLASY registration number:**

INPLASY 202130102.

## 1 Introduction

The development of gastric cancer (GC) is a multi-step process, in which gastric precancerous lesions (GPL) include chronic atrophic gastritis (CAG) with intestinal metaplasia (IM) and / or dysplasia (Dys).^[1]^ Studies have shown that the pathogenesis of GPL is complicated, which is mainly related to eating habits, *Helicobacter pylori* (Hp) infection, gastric mucosal protective dysfunction, duodenal fluid reflux, autoimmunity and genetic factors.^[2–5]^ According to the latest report of International Agency for Research on Cancer, CAG accounts for 23.6% of the population of patients with chronic gastritis, and the annual canceration rate is 0.5% to 1%.^[6]^ The study showed that 1592 patients with CAG were followed up by gastroscopy for more than 3 times in 3 to 20 years, of which 349 cases (21.92%) were diagnosed as dysplasia by histopathology, 23 cases (1.44%) developed into gastric cancer, and the incidence of CAG with IM increased year by year.^[7]^ It has the characteristics of high morbidity, high metastasis rate, high mortality rate, and low early diagnosis rate, which is a serious threat to human health.^[8]^

Even if there is a possibility of early recognition and treatment in GC, most patients tend to be diagnosed at a late stage and miss the best time for treatment. Therefore, early screening of GPL and taking effective therapeutic measures are an important part of secondary prevention of gastric cancer.^[9–11]^ In the light of present situation, Western medicine mainly starts with symptomatic treatment, mainly delaying the transition to GC by means of inhibiting gastric acid secretion and protecting gastric mucosa,^[12]^ supplementing vitamins or folic acid,^[13]^ using COX-2 inhibitor,^[14]^ eradicating Hp,^[12,15]^ and so on. However, it is not effective and easy to relapse, and can not reverse the pathological changes of gastric mucosa.^[16–18]^

With the deepening of clinical research, traditional Chinese medicine (TCM) has played an obvious advantage in the treatment of gastric precancerous lesions, which is economical and safe, effective, multitarget and less side effects, especially in reversing intestinal metaplasia, dysplasia, and even low-grade intraepithelial neoplasia.^[19]^ Banxia Xiexin Decoction (BXD)^[20]^ is a classical formula from Shang-Han-Lun which is one of the earliest books of TCM clinical practice. BXD consists of Banxia (Rhizoma Pinelliae), Huangqin (Radix Scutellariae), Ganjiang (Rhizoma Zingiberis), Renshen (Radix Ginseng), Zhigancao (Radix Glycyrrhizae Uralensis), Huanglian (Rhizoma Coptidis), Dazao (Fructus Jujubae). The prescription has the effect of harmonizing liver and spleen, dissipating distension syndrome, and dispersing nodule, enriching qi, and nourishing Yin.^[21]^ Modern studies have shown that the prescription has the effects of bi-directional regulation of stomach and intestines, clearance of Hp, protection of gastric mucosa, antiinflamation and enhancement of immunity.^[22–26]^ In recent years, many randomized controlled trials (RCTs), have been carried out in the treatment of gastric precancerous lesions with BXD. However, there are differences in the research programs and efficacy of clinical trials, and the results are uneven. Therefore, it is urgent to summarize them by systematic evaluation, so as to provide evidence-based medicine evidence for clinical application.

## 2 Objectives

The aims are:

1. to explore the efficacy of BXD for GPL and
2. to provide up-to-date evidence for clinical of GPL.

## 3 Methods

### 3.1 Study registration

The protocol of our study is conducted in strict accordance with the PRISMA-P guidelines and the Cochrane Handbook.^[27,28]^ This protocol has been registered on INPLASY (registration number: INPLASY 202130102: https://inplasy.com/inplasy-2021-3-0102/).

### 3.2 Inclusion criteria

#### 3.2.1 Type of studies

This study will include all relevant RCTs without language restrictions.

#### 3.2.2 Type of participants

Patients with chronic atrophic gastritis or with intestinal metaplasia or dysplasia diagnosed by gastroscopy, pathological examination and laboratory examination have clinical manifestations such as long-term dyspepsia, anorexia, fatigue, epigastric distension, emaciation, anemia, and so on. Positive for Hp. The patients were regardless of age, sex, or race.

#### 3.2.3 Type of interventions

BXD is used in the treatment of precancerous lesions of gastric cancer in the form of decoction, powder, capsule, and so on.

#### 3.2.4 Type of comparators

Oral routine western medicine with variable dosage form.

#### 3.2.5 Types of outcome measures

The total effective rate of clinical remission, the effective rate of gastroscopy, the clearance rate of Hp and the effective rate of pathological examination.

### 3.3 Exclusion criteria

1. Exclusion of taboos in drug use, severe mental disorders, severe liver function damage, renal function damage and blood diseases, severe digestive system diseases, and pregnant and lactating women;
2. Compilation of meetings with only abstracts;
3. Repeated publication of the same author, select the study with the highest quality and the largest sample size;
4. The literature with incomplete data or unable to obtain data.
5. Animal experiments, reviews, case reports, and other literature.

### 3.4 Search methods for identification of studies

We will conduct a comprehensive data retrieval, and the electronic databases will include PubMed, Embase, Cochrane Library, WangFang Database, China National Knowledge Infrastructure, Chinese Scientific Journal Database, Chinese Biomedical Literature Database, from establishment to March 2021. The key words include “gastric precancerous lesions,” “gastric precancerous condition,” “Gastritis, Atrophic,” “chronic atrophic gastritis” “Intestinal metaplasia,” “Dysplasia,” “Banxia xiexin decoction,” “Ban-Xia-Xie-Xin-Tang.” An equivalent translation of the same search terms will be used to search in the Chinese databases. We will also manually search unpublished studies and references. The specific search strategy of PubMed is provided in Table 1.

**Table 1:** Search strategy used in PubMed database.

Order	Search items
#1	(((((Gastritis, Atrophic[MeSH Terms]) OR (Atrophic Gastritides)) OR (Atrophic Gastritis)) OR (Gastritides, Atrophic)) OR (chronic atrophic gastritis)) OR (((Dysplasia) OR (Intestinal metaplasia)) OR ((gastric precancerous lesions) OR (gastric precancerous condition)))
#2	((((((((banxia xiexin decoction) OR (BXXXT)) OR (BXD)) OR (Ban-Xia-Xie-Xin-Tang)) OR (Pinelliae decoction for purgingstomach-fire)) OR (Banxia xiexin soup)) OR (pinellia heart-sedating granules)) OR (Banxiaxiexin)) OR (cold-heat complex syndrome)
#3	randomized controlled trial[Publication Type] OR randomized[Title/Abstract] OR placebo[Title/Abstract]
#4	#1 AND #2 AND #3

### 3.5 Studies selection

**Figure 1:**
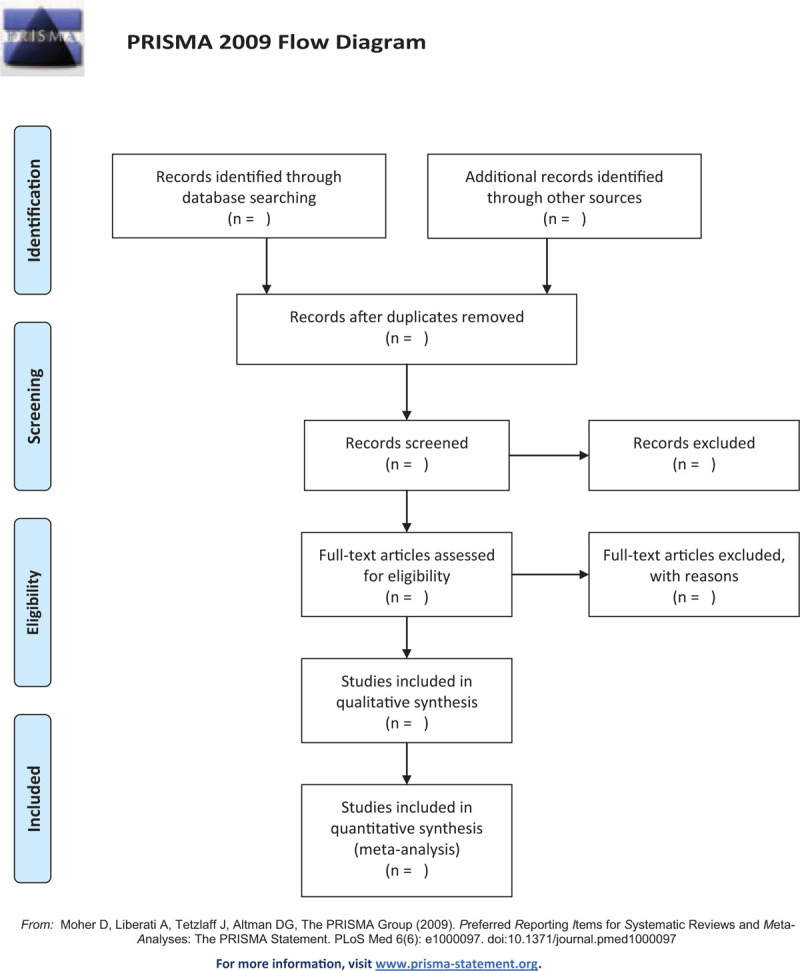
Flowchart of literature selection.

Endnote V.9.1 software will eliminate duplicate studies from all the obtained literatures. The unqualified studies in the remaining articles will be eliminated by 2 reviewers by reading the title and abstract. Then, 2 reviewers will read the full text to determine the final included studies. If the significant information of the article is incomplete, we will contact the author. In all the processes, the researchers will operate independently. When 2 reviewers have disagreements, the decision will be made by the third researcher. The selection process will be showed in a PRISMA flow diagram (Fig. 1).

### 3.6 Data extraction and management

We will establish a data extraction table, which will be used by 2 researchers to extract data from qualified literature. The specific contents will include: author, publication time, participant characteristics, intervention (s), comparison (s), outcome (s), adverse events, and some relevant features. If the significant information of the article is incomplete, we will contact the author. In case of disagreements, the third researcher will be consulted.

### 3.7 Assessment of the methodological quality

The Cochrane risk assessment tool will be used by us to evaluate the methodological quality of qualified RCTs.^[28]^ It includes 7 items: random sequence generation, allocation concealment, blinding of participants and caregivers, blinding of outcome assessors, incomplete outcome data, selective outcome reporting, and other bias. The evaluation result of each item will be “high risk,” “low risk,” or “unclear risk.” The assessment will be completed by 2 reviewers, and disagreements will be handed over to the third reviewer for the final decision.

### 3.8 Measures of treatment effect

Mean difference) or standard mean difference will be used for continuous outcomes with 95%confidence intervals (CIs). Dichotomous outcomes will be summarized by risk ratio with 95% CIs.

### 3.9 Dealing with missing data

When there are missing data, we will attempt to contact the original authors of the study to obtain the relevant missing data. Important numerical data will be carefully evaluated. If missing data cannot be obtained, an imputation method will be used. For studies having insufficient data to enter in the meta-analysis, even after contacting the authors, we will report the results qualitatively.^[29]^

### 3.10 Assessment of heterogeneity

Statistical heterogeneity should be evaluated by Chi-Squared tests and *I*^2^ statistic. The results of the *I*^2^ statistic, which determine the using of fixed-effects model or random-effects model, cover unimportant heterogeneity (0%–40%), moderate heterogeneity (30%–60%), substantial heterogeneity (50%–90%), and considerable heterogeneity (75%–100%). A random-effect model or subgroup analysis should be used when there exists significant heterogeneity.

### 3.11 Data synthesis

Data synthesis will be completed using RevMan5.3.5 software. When *I*^2^ < 50%, we will choose the fixed effects model; Otherwise, the random effects model will be selected. The forest plots will present the results of the meta-analyses. We will conduct descriptive analysis, when the results are not suitable for consolidation. When more than 10 studies are included, we will use the funnel plot to assess publication bias.

### 3.12 Subgroup analysis

If necessary, subgroup analyses will be performed according to the different types of participant characteristics, treatment methods, treatment frequency, and so on.

### 3.13 Sensitivity analysis

When there is significant heterogeneity, we will conduct a sensitivity analysis. We will determine the robustness of the results by excluding low-quality studies.

### 3.14 Grading the quality of evidence

Two reviewers will independently use the Grading of Recommendations Assessment, Development and Evaluation, which evaluates the quality of evidence as “high,” “moderate,” “low,” or “very low,” to assess the quality of evidence.^[30]^

### 3.15 Ethics and dissemination

In this study, no individual data from participants will be involved, so ethics approval is not required. This systematic review will be published through peer-reviewed journal.

## 4 Discussion

As an effective treatment of GPL, TCM can effectively reduce the clinical symptoms, improve the quality of life and delay the life cycle of patients. BXD has a good curative effect on the syndrome of loss of transportation of spleen and stomach, abnormal ascending and descending of qi, mixed cold and heat, etc. As far as it goes, the TCM research on GPL lacks an overall review of the original literature, which makes it difficult for researchers to grasp this field. Therefore, the purpose of this paper is to comprehensively search the literature related to BXD in the treatment of GPL, and evaluate the overall efficacy and safety of BXD in the treatment of GPL through qualitative or quantitative analysis, so as to provide corresponding guidance for clinical decision-making.

## Author contributions

**Conceptualization:** Ying Yi, Ziyi Hu.

**Data curation:** Ying Yi, Hong Li, Hengyi Zhang.

**Formal analysis:** Renliang Li, Lisha Chen, Wanting Liu.

**Investigation:** Mengwen Wu, Lisha Chen, Renliang Li.

**Methodology:** Hong Li, Mengwen Wu, Wanting Liu.

**Software:** Lisha Chen, Hengyi Zhang.

**Supervision:** Ziyi Hu, Renliang Li, Hengyi Zhang.

**Writing – original draft:** Ying Yi, Renliang Li, Hengyi Zhang.

**Writing – review & editing:** Lisha Chen, Hong Li, Wanting Liu, Mengwen Wu, Ziyi Hu.
